# Intakes of Dairy Products and Calcium and Obesity in Korean Adults: Korean National Health and Nutrition Examination Surveys (KNHANES) 2007-2009

**DOI:** 10.1371/journal.pone.0099085

**Published:** 2014-06-10

**Authors:** Hae-Jeung Lee, Jang-ik Cho, Hye-Seung H. Lee, Cho-il Kim, Eunyoung Cho

**Affiliations:** 1 Department of Food & Nutrition, Eulji University, Seongnam-si, Gyeonggi-do, South Korea; 2 Department of Epidemiology and Biostatistics, Case Western Reserve University, Cleveland, Ohio, United States of America; 3 Channing Division of Network Medicine, Department of Medicine, Brigham and Women's Hospital and Harvard Medical School, Boston, Massachusetts, United States of America; 4 Department of Health Industry & Policy, Korea Health Industry Development Institute, Cheongwon-gun, Chungcheongbuk-do, South Korea; 5 Department of Dermatology, The Warren Alpert Medical School of Brown University, Providence, Rhode Island, United States of America; University of Louisville, United States of America

## Abstract

**Background:**

The possible effects of dairy product intake against obesity have been suggested in animal studies; however, the association is still not well established in epidemiological studies. Few studies in Asian countries with relatively low intake of dairy products exist.

**Objective:**

We investigated the association between dairy products and calcium intake and obesity in Korean population with relatively low intake of dairy products.

**Subjects and Methods:**

Our study population consisted of adults (n = 7173) aged 19–64 among participants of the 2007, 2008 and 2009 Korean National Health and Nutrition Examination Survey who had not made any attempt of intentional weight loss. Dietary intake data from food frequency questionnaire (FFQ) and 24-hour recall were used. Dairy products included milk and yogurt in the FFQ. Obesity was defined as BMI≥25 kg/m^2^.

**Results:**

Higher frequency of dairy product intake was associated with a reduced prevalence of obesity (OR = 0.63; 95% CI = 0.45–0.89 for ≥2 times/day vs. ≤1 time/month; p for trend = 0.003) using the intake data from FFQ. Similarly, high frequency of milk or yogurt intake had an inverse association with obesity. The association between milk and yogurt intake and obesity was similar when the intake from 24-hour recall was examined. Higher calcium intake from dairy products as well as total dietary calcium intake was associated with a decreased prevalence of obesity (OR = 0.83; 95% CI = 0.71–0.98 for highest vs. lowest quintile of dairy calcium intake; p for trend = 0.02, OR = 0.78; 95% CI = 0.64–0.94 for highest vs. lowest quintile of total calcium intake; p for trend = 0.04). The associations appeared to be stronger in women than in men.

**Conclusion:**

These results suggest that high consumption of dairy products is associated with a lower prevalence of obesity and that calcium in dairy products may be one of the components contributing to the association. Further longitudinal studies are warranted to replicate our findings.

## Introduction

It has been suggested that dairy products may reduce the risk of obesity through the properties of their individual components including calcium, branched chain amino acids, conjugated linoleic acid, protein, vitamin D, and medium-chain fatty acids [1]. Recent reviews suggested modest inverse association between dairy product intake and obesity [2], [3]. Studies on dairy product intake and obesity have been conducted in Western countries but are scarce in Asian countries, where consumption of dairy products is often much lower than that of Western countries. According to the 2009 Korea National Health and Nutrition Examination Survey (KNHANES), the mean frequencies of milk and yogurt intake of Koreans were 3.0 and 1.2 times/week, respectively [4].

One of the major components of dairy products which may be related to obesity is calcium. Although recent reviews reported that dietary calcium intake could lead to weight loss [1], [5], few studies exist in Asian countries on this issue [6].

Thus, we examined the association between consumption of dairy products and obesity, and whether calcium intake is negatively related to obesity in Korean adults.

## Subjects/Methods

### Subjects and exclusion criteria

KNHANES was conducted under the National's Health Promotion Act in Korea, designed to gather data on health status, attitudes and behavior towards health, diet, and nutrition of Korean population. The survey was conducted in four rounds (1998, 2001, 2005, and 2007–2009). The fourth round, from which the data for our analyses were collected, was conducted throughout the year to avoid seasonal bias in diet. The participants of the 4th KNHANES were selected using a rolling sampling method designed to represent the Korean population since 2007 [7]. KNHANES consisted of a health interview, health examination, and nutrition survey.

In the 2007, 2008 and 2009 KNHANES, 4594, 9744, and 10533 individuals participated, respectively. After we excluded subjects under 19 and over 65 years old, the number of participants was reduced to 14,334. In order to reduce any potential bias, we have excluded subjects who reported change in their diet to lose weight (n = 3,659), which resulted in 14,334 adults. Then, we excluded participants who were either pregnant (n = 123), who did not have dietary intake data (n = 1,924), whose caloric intake was rather extreme of either <500 or 6000 kcal/day (n = 103), or who did not have anthropometric data to calculate BMI (n = 869). We excluded those who were underweight (n = 535) because they could have a causal-effects. Subsequently, we had 7,173 subjects in final analysis (men = 3,400, and women  = 3,773).

The study was conducted in accordance with the Ethical Principles for Medical Research Involving Human Subjects, as defined by the Helsinki Declaration. All the study subjects were provided with written informed consent for the survey. Moreover, de-identified data were used in the study.

### Dietary assessment and dairy product intake

Extensive interviews for dietary intake were conducted at participant's home. Ten to twelve nutritionists underwent a regular one-week intensive training program at the beginning of survey year and were assigned to 40 to 50 sited to conduct nutrition survey. Each nutrition survey team consisted of two nutritionists. Moreover, quality control on interview at field was conducted throughout the survey by the Center for Nutrition Policy and Promotion at the Korea Health Industry Development Institute.

Dietary assessments consisted of a qualitative food frequency questionnaire (FFQ) for the past year and one-day-24-hour recall. The FFQ consisted of 63 commonly consumed food items in Korea with frequency of food intake in 9 categories (almost never, 6–11 times/year, 1 time/month, 2–3 times/month, 1 time/week, 4–6 times/week, 1 time/day, 2 times/day, and 3 times/day). Cheese was not included in the FFQ because cheese intake is very low in Korea. Therefore, we defined dairy product as a combination of milk and yogurt in our analysis. We categorized intake of milk and yogurt as none, ≤ l/month, >1/month- ≤1/week, >1/week - <1/day, and ≥1/day. The intake category of dairy products was categorized as ≤l/month, >1/month-≤1/week, >1/week-<3/week, ≥3/week-<1/day, ≥1/d-<2/day, and ≥2/day.

We also examined dairy product intake from 24-hour recall. We extracted milk intake data and separated them into low-fat (<2%), medium-fat (2-< 3.4%), and high-fat (≥3.4%) according to fat content (g/100 g). The number of subjects consuming low-fat milk was very low (n = 94) so that we combined low- and medium-fat as “low or medium-fat” (<3.4%). In case of yogurt, we were able to categorize it into liquid vs. semi-solid type in the 24-hour recall data. Intakes of total calcium, non-dairy calcium, and dairy calcium were evaluated using 24-hour recall data. Non-dairy yogurt was not included.

### Anthropometric measurements

Weight and height were measured by well-trained medical professionals. Standing height was measured when the subject faced directly ahead with shoes off, feet together, arms by the sides, and heels, buttocks, and upper back in contact with the wall. The unit of height was measured in centimeters with one decimal point using SECA 225 (Germany, SECA). Weight was measured using GL-6000-20 scale (Cass Korea) in kilograms with one decimal point. BMI was calculated as weight (kg)/height (m^2^).

We defined obesity as a BMI ≥25 kg/m^2^, consistent with the proposals of the Asia-Pacific region of World Health Organization (WHO) [8] and the criteria by the Korea Centers for Disease Control and Prevention (KCDC).

### Statistical analysis

Baseline characteristics of dairy product intakes in quintile groups were analyzed. Means and standard errors (SEs) of continuous variables were calculated. The Proportions of each covariate in the quintile groups for each group was calculated. P value was calculated by using generalized linear model (GLM) for continuous variables and Chi-square test for categorical variables.

We conducted logistic regression to assess the associations between dairy product intake and obesity using data from 24-hour recall and FFQ. In 24-hour recall data, 212.0 g (approximately 1 glass of milk) was the median intake among those who drank milk, which used it as a cutoff point. For calcium, we used quintiles for intake of total calcium and calcium from non-dairy sources. For dairy calcium, we categorized into 3 categories (0, <214 mg/day, and ≥214 mg/day). 214 mg was the median of dairy calcium intake among those who consumed dairy product, which used it as a cutoff point.

All the models were adjusted for age and sex. We conducted multivariable logistic regression analyses for the association between dairy product intake and obesity. The covariates such as education, income, smoking, alcohol drinking, and physical activity were associated with the risk of obesity in the age and sex logistic regression analysis (P<0.10). Energy intake is well-know the risk factor of obesity. Therefore, the multivariable model was additionally adjusted for income (lowest, low, high, highest; defined by KCDC), education (elementary school, middle school, high school, college or higher), alcohol drinking (continuous), smoking status (non-smoker, past smoker, current smoker and <1 pack/day, current smoker and ≥1 packs/day), physical activity (no-exercise or irregular walking, regular walking, regular moderate-level activity, regular vigorous-level activity), survey year (2007, 2008, and 2009), and energy intake (continuous). For the secondary analysis of dairy products, we further adjusted for calcium intake because calcium intake could potentially reduce the risk of being obesity [1], [5].In addition, we conducted a stratified analysis by gender. To assess whether there was any linear association between dairy products or calcium intake and obesity, tests for linear trends were performed by treating the median value of each intake category of dairy product or calcium as a continuous variable. All analyses were performed using SAS statistical software (version 9.2; SAS Institute Inc., Cary, NC).

## Results

The characteristics of the study population according to the frequency of dairy product intake assessed by FFQ are presented in Table 1. Participants who consumed dairy products more frequently tended to be younger and to have lower BMI and higher calcium intake. They were also more likely to be female and non-smokers and to have higher education and income.

**Table 1:** Baseline characteristics according to frequency of dairy product intake among the adults in the Korea National Health and Nutrition Examination Survey (KNHANES) 2007–2009.

Frequency of dairy product intake		≤ 1month	>1/month - ≤1/wk	>1/wk - <3/wk	≥3/wk - <1/day	≥1/day - <2/day	≥2/day	P value
		(n = 1476)	(n = 1226)	(n = 1441)	(n = 1115)	(n = 1669)	(n = 246)	
Mean±S.E								
Age (age)		49.2±0.3	45.9±0.3	43.1±0.3	41.0±0.3	41.8±0.3	39.5±0.7	<.0001
Body mass index (BMI, kg/m^2^)		23.9±0.1	23.8±0.1	23.5±0.1	23.8±0.1	23.4±0.1	23.4±0.2	<.0001
Energy (kcal)		1897.0±22.0	1947.4±21.6	1953.6±17.9	2020.7±23.1	2034.4±18.5	2197.2±74.3	<.0001
Calcium (mg)		432.9±8.5	464.4±8.5	474.6±7.4	529.0±1040	583.5±8.6	687.3±29.2	<.0001
Alcohol intake(servings/wk)		6.6±0.3	7.5±0.3	6.6±0.3	5.7±0.3	5.7±0.2	6.2±0.8	<.0001
Percent (%)								
BMI ≥25 (kg/m^2^)		33	30	27	31	27	23	<.0001
Female		48	48	48	55	61	64	<.0001
Smoking	Non-smoker	55	54	55	56	56	55	<.0001
	Past smoker	15	18	19	18	20	20	
	Current, < 1pack per day	14	14	13	14	13	16	
	Current, ≥ packs per day	16	14	13	12	11	9	
Physical activity	Do not exercise/walk sometimes	42	43	45	41	40	40	
	Regularly walk	27	29	28	31	30	29	0.0170
	Regularly moderate-level activity	11	10	9	9	11	8	
	Regularly vigorous-level activity	17	17	17	19	18	22	
Education	Elementary school	25	20	16	15	15	15	<.0001
	Middle school	15	14	16	12	12	12	
	High school	33	37	33	34	33	32	
	College or higher degree	27	29	35	39	40	42	
Income	1^st^ (Lowest)	35	29	25	22	21	22	
	2^nd^	26	26	25	24	25	23	<.0001
	3^rd^	21	23	24	26	26	27	
	4^th^ (Highest)	16	19	24	28	28	28	

Frequency of dairy product intake includes milk and yogurt items.

P-value was calculated by GLM for continuous variables and Chi-square test for categorical variables.

Table 2 presents the odds ratio of obesity according to the level of dairy product consumption from FFQ. We found that higher dairy product intake was associated with a lower prevalence of obesity (OR = 0.63; 95% CI = 0.45–0.89 for ≥2 times/day vs. ≤1 time/month; p for trend  = 0.003). Similarly, intakes of milk and yogurt were each associated with a lower prevalence of obesity (milk: OR = 0.81; 95% CI = 0.68–0.96 for ≥1 time/day vs. 0 [p for trend = 0.002]; yogurt: OR = 0.77; 95% CI = 0.59–1.00 for ≥1 time/day vs. 0 [p for trend = 0.01]). In a secondary analysis, we further adjusted for calcium intake in the multivariate models; the results for dairy product, milk, and yogurt remained similar. In a stratified analysis by gender, intakes of dairy products and milk were associated with obesity only in women. Yogurt intake was not associated with obesity in either men or women (Table 3).

**Table 2:** Age- and gender-adjusted and multivariate-adjusted ORs and 95%CIs for obesity according to the frequency of dairy products intake using food frequency questionnaire for 1 year among the adults in the Korean Health and Examination Survey (KNHANES).

Frequency	Median (time/wk)	No of participants(cases)	Age, sex -adjusted OR	Multivariate OR	Multivariate+calcium OR
*Dairy products*					
≤1/month	0	1476(509)	Reference	Reference	Reference
> 1/month - ≤1/wk	0.8	1226(386)	0.92 (0.78–1.08)	0.92 (0.78–1.09)	0.93 (0.79–1.10)
>1/wk - <3/wk	2.5	1441(395)	0.79 (0.70–0.96)	0.82 (0.70–0.970)	0.83 (0.70–0.97)
≥3/wk - <1/day	5.2	1115(328)	0.94 (0.79–1.11)	0.99 (0.83–1.18)	1.00 (0.75–1.19)
≥1/day - <2/day	8	1669(414)	0.75 (0.64–0.88)	0.79 (0.67–0.93)	0.80 (0.68–0.95)
≥2/day	14	246(49)	0.59 (0.42–0.83)	0.63 (0.45–0.89)	0.65 (0.46–0.91)
	P for trend		<.0001	0.003	0.007
*Milk*					
0	0	1445(464)	Reference	Reference	Reference
≤1/month	0.25	931(304)	1.04 (0.87–1.24)	1.05 (0.88–1.25)	1.05 (0.88–1.26)
>1/month - ≤1/wk	1	1440(435)	0.97 (0.83–1.14)	0.97 (0.83–1.14)	0.98 (0.83–1.15)
>1/wk - <1/day	2.5	1735(485)	0.92 (0.79–1.08)	0.95 (0.81–1.11)	0.96 (0.82–1.12)
≥ 1/day	7	1559(372)	0.78 (0.66–0.92)	0.81 (0.68–0.96)	0.82(0.69–0.97)
	P for trend		0.0004	0.002	0.006
*Yogurt*					
0	0	2594(861)	Reference	Reference	Reference
≤1/month	0.25	1451(400)	0.84 (0.72–0.97)	0.86 (0.75–1.00)	0.87 (0.75–1.00)
>1/month - ≤1/wk	1	1689(445)	0.83 (0.72–0.96)	0.89 (0.77–1.03)	0.89 (0.77–1.03)
>1/wk - <1/day	2.5	1008(267)	0.86 (0.73–1.02)	0.92 (0.77–1.09)	0.92 (0.78–1.10)
≥1/day	7	368(87)	0.70 (0.54–0.91)	0.77 (0.59–1.00)	0.78 (0.60–1.01)
	P for trend		0.01	0.01	0.05

Multivariate models were adjusted for age (continuous), gender, survey year (2007/2008/2009), education (elementary school, middle school, high school, college or higher degree), smoking (non-smoker, past smoker, current and < 1pack per day, current and ≥1pack per day), alcohol intake (never drink, <1drink/month, ≥ 1drink/mo & <2drinks/wk and for female <5 glasses/drink, for male <7 glasses/drink; ≥ 2 drinks/week and for female ≥ 5 glasses/drink, for male ≥7 glasses/drink), physical activity (no-exercise or irregularly walking, regularly walking, regular moderate-level activity, regular vigorous-level activity), income (1^st^(lowest), 2^nd^, 3^rd^, 4^th^(highest)), energy intake (continuous).

**Table 3:** Multivariate and calcium-adjusted ORs and 95%CIs for obesity according to the frequency of dairy products intake using food frequency questionnaire for 1 year among the adults in the Korean Health and Examination Survey (KNHANES) by gender.

			MEN			WOMEN	
Frequency	Median (per week) men/women	No of participant (cases)	Multivariate OR	Multivariate+calcium OR	No of participants (cases)	Multivariate OR	Multivariate+calcium OR
*Dairy products*							
≤1/month	0/0	761(269)	Reference	Reference	715 (240)	Reference	Reference
> 1/month - ≤1/wk	0.6/0.8	641(228)	0.96 (0.77–1.21)	0.97 (0.77–1.21)	585 (158)	0.86 (0.67–1.11)	0.87 (0.68–1.11)
>1/wk - < 3/wk	2.0/2.5	750(229)	0.79 (0.63–0.99)	0.79 (0.63–0.99)	691 (166)	0.83 (0.65–1.06)	0.83 (0.65–1.06)
≥3/wk - <1/day	5/5.3	503(174)	0.98 (0.76–1.25)	0.98 (0.77–1.26)	612 (154)	0.99 (0.76–1.27)	0.99 (0.77–1.28)
≥1/day - < 2/day	7.6/7.5	657(223)	0.95 (0.76–1.20)	0.96 (0.76–1.22)	1012 (191)	0.66 (0.52–0.83)	0.68 (0.53–0.85)
≥2/day	14/14	88(23)	0.68 (0.40–1.11)	0.68 (0.41–1.14)	158 (26)	0.62 (0.39–0.99)	0.64 (0.40–1.02)
	P for trend		0.46	0.53		0.002	0.003
*Milk*							
0	0/0	706(464)	Reference	Reference	739 (464)	Reference	Reference
≤1/month	0.25/0.25	491(304)	1.05 (0.82–1.34)	1.05 (0.82–1.35)	440 (304)	1.04 (0.80–1.36)	1.04 (0.80–1.36)
>1/month - ≤ 1/wk	1/1	763(435)	1.01 (0.81–1.34)	1.01 (0.81–1.26)	677 (435)	0.91 (0.71–1.16)	0.91 (0.71–1.16)
>1/wk - <1/day	2.5/2.5	821(485)	0.91 (0.73–1.14)	0.92 (0.73–1.14)	914 (485)	1.02 (0.81–1.28)	1.02 (0.81–1.29)
≥ 1/day	7/7	584(372)	1.01 (0.79–1.29)	1.02(0.80–1.31)	975 (372)	0.68 (0.53–0.86)	0.69 (0.54–0.88)
	P for trend		0.91	0.99		0.0002	0.0004
*Yogurt*							
0	0/0	1260(463)	Reference	Reference	1334 (398)	Reference	Reference
≤1/month	0.25/0.25	732(223)	0.74 (0.61–0.91)	0.74 (0.61–0.91)	719 (177)	0.98 (0.79–1.22)	0.99 (0.80–1.23)
>1/month - ≤1/wk	1/1	784(244)	0.79 (0.65–0.97)	0.80 (0.65–0.98)	905 (201)	0.97 (0.78–1.19)	0.97 (0.79–1.20)
>1/wk - < 1/day	2.5/2.5	420(153)	1.00 (0.78–1.27)	1.00 (0.79–1.28)	588 (114)	0.87 (0.67–1.11)	0.87 (0.68–1.12)
≥1/day	7/7	169(50)	0.73 (0.51–1.05)	0.73 (0.51–1.05)	199 (37)	0.81 (0.55–1.20)	0.82 (0.55–1.20)
	P for trend		0.43	0.45		0.18	0.21

Multivariate models were adjusted to the covariables except gender as denoted in Table 2.

When we examined the intake from the 24-hour-recall, there was an inverse association between intake of dairy products and obesity (Table 4). When we stratified milk by fat content, only low- and medium-fat milk (less than 3.4% fat) had inverse associations with obesity (OR = 0.80; 95% CI = 0.60–1.00 for >212 g/day vs. non-consumer group; p for trend = 0.03, OR = 0.79; 95% CI = 0.67–0.93 for consumer vs. non-consumer group). Neither liquid nor semi-solid type yogurt intake showed any association with the obesity.

**Table 4:** Age- and gender-adjusted and multivariate-adjusted ORs and 95%CIs for obesity according to the amount of dairy products intake per day using 1day-24hr-recall among the adults in the Korean Health and Examination Survey (KNHANES).

Variables	Category	Median	No of participants(cases)	Age, sex -adjusted OR	Multivariate OR
	(g/day)	(g/day)			
	0	0	5612 (1694)	Reference	Reference
*Dairy Products*	>0-< 212.0	122.4	506 (130)	0.87 (0.71–1.08)	0.91 (0.74–1.12)
*(milk and yogurt)*	212.0	212.0	511 (112)	0.71 (0.57–0.89)	0.73 (0.58–0.91)
	> 212.0	381.6	544 (145)	0.92 (0.75–1.12)	0.93 (0.76–1.15)
		P for trend		0.06	0.07
	>0	212.0	1561 (387)	0.83 (0.73–0.95)	0.85 (0.75–0.98)
	0	0	5876 (1761)	Reference	Reference
*Total Milk*	>0- < 212.0	108.2	277 (70)	0.88 (0.66–1.16)	0.91 (0.69–1.21)
	212.0	212.0	537 (122)	0.75 (0.61–0.92)	0.77 (0.62–0.95)
	> 212.0	381.6	483 (128)	0.92 (0.74–1.14)	0.94 (0.76–1.16)
		P for trend		0.05	0.10
	>0	212.0	1297 (320)	0.84 (0.73–0.96)	0.86 (0.74–0.99)
	0	0	5876 (1761)	Reference	Reference
	>0- <212.0	124.6	208 (55)	0.93 (0.67–1.27)	0.96 (0.70–1.32)
*Low or Medium fat milk*	212.0	212.0	381 (78)	0.65 (0.50–0.84)	0.68 (0.52–0.88)
	> 212.0	371.0	310 (75)	0.81 (0.62–1.06)	0.80 (0.60–1.00)
		P for trend		0.03	0.03
	>0	212.0	899 (208)	0.77 (0.65–0.90)	0.79 (0.67–0.93)
*High-fat milk*	0	0	5876 (1761)	Reference	Reference
	> 0	212.0	421 (118)	0.99 (0.80–1.24)	1.02 (0.81–1.28)
	0	0	6806 (1992)	Reference	Reference
	>0-<137.8	84.8	182 (43)	0.82 (0.58–1.16)	0.83 (0.59–1.18)
*Total yogurt*	≥ 137.8	169.5	185 (46)	0.83 (0.59–1.17)	0.86 (0.61–1.21)
		P for trend		0.15	0.23
	>0	137.8	367 (89)	0.82 (0.64–1.06)	0.85 (0.66–1.09)
*Liquid yogurt*	0	0	6806 (1992)	Reference	Reference
	> 0	68.9	138 (36)	0.90 (0.62–1.34)	0.89 (0.60–1.31)
*Semi-solid yogurt*	0	0	6806 (1992)	Reference	Reference
	> 0	169.5	235 (54)	0.77 (0.56–1.05)	0.81 (0.59–1.11)

The group of intake >0 was excluded for p-for trend test in each variable.

Multivariate models were adjusted for the same covariables as denoted in Table 2.

In addition, we explored whether calcium, one of the important nutrients of dairy products, has a protective effect against obesity (Table 5). After adjusting for potential confounders, higher calcium intake from dairy products as well as total calcium intake were associated with a lower prevalence of obesity (dairy calcium: OR = 0.83; 95% CI = 0.71–0.98 for ≥214 mg/d vs. 0 mg/d; p for trend  =  0.02, total calcium; OR = 0.78; 95% CI = 0.64–0.94 for highest vs. lowest quintiles; p for trend = 0.04). Association of obesity with non-dairy sources of calcium intake was not significant. Total dietary calcium and dairy calcium intake were inversely associated with obesity in women but not in men, while non-dairy calcium intake was not associated with risk of obesity in both men and women (Table 6).

**Table 5:** Age- and gender-adjusted and multivariate-adjusted ORs and 95%CIs for obesity according to the amount of calcium intake derived from different sources per day using 1day-24hr recall among the adults in the Korean Health and Examination Survey (KNHANES).

	Median (mg/day)	No of participants	Age, sex -adjusted OR	Multivariate OR
*Total calcium intake*				
Quintile 1	196.8	1434(434)	Reference	Reference
Quintile 2	319.5	1435(385)	0.80 (0.68–0.95)	0.80 (0.68–0.95)
Quintile 3	433.0	1435(427)	0.90 (0.76–1.05)	0.87 (0.74–1.03)
Quintile 4	584.3	1435(411)	0.84 (0.71–0.99)	0.81 (0.68–0.96)
Quintile 5	880.4	1434(424)	0.84 (0.72–0.99)	0.78 (0.64–0.94)
	P for trend		0.13	0.04
*Calcium from dairy products*				
0	0	5220(1597)	Reference	Reference
<214.0*	93.6	976(245)	0.85 (0.72–0.99)	0.86 (0.74–1.02)
≥214.0	297.3	977(239)	0.81 (0.69–0.95)	0.83 (0.71–0.98)
	P for trend		0.01	0.02
*Calcium from other sources*				
Quintile 1	175.9	1434(404)	Reference	Reference
Quintile 2	281.1	1435(390)	0.89 (0.76–1.05)	0.89 (0.75–1.06)
Quintile 3	376.2	1435(402)	0.89 (0.76–1.05)	0.88 (0.74–1.05)
Quintile 4	502.4	1435(433)	0.94 (0.80–1.12)	0.92 (0.77–1.10)
Quintile 5	750.0	1434(452)	0.94 (0.80–1.11)	0.89 (0.74–1.08)
	P for trend		0.88	0.45

Multivariate models were adjusted to the covariables as denoted in Table 2.

*Median value among those who had intake of dairy calcium in 24-hr recall data.

**Table 6:** Age-adjusted and multivariate-adjusted ORs and 95%CIs for obesity according to the amount of calcium intake derived from different sources per day using 1day-24hr recall among the adults in the Korean Health and Examination Survey (KNHANES) by gender.

		Median	MEN				WOMEN			
Intake, mg/day		(men/women, mg/day)	No. of participants	No. of case	age-adjusted	Multivariate OR	No of participants	No of case	age-adjusted	Multivariate OR
*Total calcium intake*	Quintile 1	202.3/194.9	435	155	reference	reference	999	279	reference	reference
	Quintile 2	321.4/317.9	605	179	0.76 (0.58–0.98)	0.71 (0.54–0.93)	830	206	0.89 (0.72–1.10)	0.90 (0.72–1.12)
	Quintile 3	436.2/430.7	716	251	0.97 (0.76–1.25)	0.87 (0.67–1.13)	719	176	0.85 (0.68–1.06)	0.87 (0.69–1.10)
	Quintile 4	590.1/577.0	761	263	0.95 (0.74–1.22)	0.85 (0.65–1.11)	674	148	0.76 (0.61–0.97)	0.79 (0.61–1.01)
	Quintile 5	890.6/871.0	883	298	0.92 (0.72–1.17)	0.80 (0.61–1.05)	551	126	0.77 (0.60–0.98)	0.76 (0.58–1.00)
		P for trend			0.78	0.54			0.02	0.04
*Calcium from dairy products*	0	0/0	2577	885	reference	reference	2643	712	reference	reference
	<214.0*	90.1/96.6	408	135	0.97 (0.78–1.22)	0.94 (0.75–1.18)	568	110	0.77 (0.61–0.97)	0.82 (0.65–1.03)
	≥214.0	318.0/283.8	415	126	0.86 (0.68–1.07)	0.86 (0.68–1.08)	562	113	0.74 (0.59–0.93)	0.79 (0.63–1.00)
		P for trend			0.18	0.18			0.01	0.05
*Calcium from other sources*	Quintile 1	178.6/175.1	388	131	reference	reference	1046	273	reference	reference
	Quintile 2	284.7/279.5	566	184	0.94 (0.71–1.23)	0.89 (0.67–1.17)	869	206	0.89 (0.72–1.10)	0.89 (0.72–1.11)
	Quintile 3	380.6/372.7	686	213	0.87 (0.67–1.14)	0.81 (0.61–1.07)	749	189	0.97 (0.78–1.20)	0.98 (0.77–1.23)
	Quintile 4	503.5/500.5	819	283	1.03 (0.79–1.32)	0.94 (0.72–1.23)	616	150	0.87 (0.69–1.10)	0.89 (0.68–1.15)
	Quintile 5	750.2/748.6	941	335	1.06 (0.83–1.36)	0.95 (0.72–1.26)	493	117	0.82 (0.64–1.05)	0.82 (0.62–1.10)
		P for trend			0.19	0.62			0.13	0.22

Multivariate models were adjusted to the covariables except gender as denoted in Table 2.

*Median value among those who had intake of dairy calcium in 24-hr recall data.

## Discussions

We found that higher intake of dairy products including milk and yogurt was associated with lower prevalence of obesity in Korean adults. Even though dairy product intake was much lower than that of Western countries, we still found strong inverse association between dairy product consumption and obesity in Korean population. Higher intake of dairy calcium as well as total calcium was associated with lower prevalence of obesity.

There have been several studies on the association between dairy product intake and obesity or weight gain in Western countries. Recent review of this issue suggested a moderately inverse association based on the studies of adolescent, adult, and elderly subjects. [2]. For adults-only studies, the results have been mixed; some studies found inverse associations [9]–[12], consistent with our results, while others found no association between dairy products and obesity [13]–[15]. For example, the CARDIA study [11] found an inverse association between dairy consumption and obesity during 10-years of follow-up, whereas the Health Professionals Follow-up Study found no association with weight gain [14]. To our knowledge, there has been no large-scale and national-level study on the association between dairy product intake and obesity in Asian populations. One small study in Japan on the relationship between dairy product intake and BMI among female college students found no association [6].

Dairy product consumption in many Asian countries including Korea used to be low but has been gradually increasing. For example, mean dairy product intake among Korean population was 2.4 g/day in 1969 and has increased to 101.2 g/day in 2009[16]. Still, the intake is much lower than that of Western countries. For example, average milk intake was approximately 67 g/day in Korea in 2005 [17] compared to 170 g/day in the U.S in 2005-2006 [18]. Nevertheless, even with dairy product intake much lower than that of Western countries, we found strong inverse association between dairy product consumption and obesity in Korean population.

Our results on dairy products intake from 24-hour recall data confirmed the findings from FFQ data and revealed that low and medium-fat milk intake but not high-fat milk intake was associated with a lower prevalence of obesity. Alternatively, consuming high-fat dairy products may lead to weight gain, whereas consuming low-fat dairy products may result in weight loss as found in a randomized crossover trial [19].

High intake of calcium was inversely associated with obesity in our population, similar to previous studies [5], [20], [21]. The potential protective mechanism of calcium is to regulate the adipocyte metabolism to depress parathyroid hormone and 1, 25-hydroxy vitamin D to decrease intracellular calcium, which may inhibit lipogenesis and stimulate lipolysis [22], [23]. Another function of calcium is to increase excretion of fecal fat [24]–[26]. We also found that calcium intake from dairy products was inversely associated with obesity, which contradicts with some studies [14], [27], while is consistent with others [28]–[30].

Besides calcium, other components of dairy products which may be responsible for the inverse association with obesity include branched chain amino acids [31], vitamin D [5], conjugated linoleic acid [32], medium-chain fatty acids [33], bioactive peptides from casein, and whey protein [34]. The association of dairy product intake and obesity was not essentially changed after adjusting for calcium intake in the multivariable models, which indicates that other components in dairy products may also contribute to the inverse relationship between dairy products and obesity although vitamin D is not universally fortified in milk in Korea.

Our secondary analysis found that there may be some gender differences in the relationship between dairy or calcium intake and obesity with stronger inverse association among female than in male, although the results in male still suggested inverse association. Because the sample size for men and women were similar, it may not be due to differences in statistical power. Similar gender difference in the association between milk consumption and change in BMI was found in a French study with significant association in women only [15]. Furthermore, the third National Health and Nutrition Examination Survey [22], HERITAGE Family study [35], and Québec Family Study [36], found an inverse association of calcium intake with body fat and BMI among women but not among men. Differences in diet and lifestyle between men and women might have contributed to the gender difference [15]. In case of yogurt intake, we did not have large number of cases in high intake group to detect an association. Thus, we shouldn't rule out the possibility that yogurt intake is also inversely related to obesity.

Our study has several strengths. First, this is one of the few first Asian studies that examined the association of dairy product intake and prevalence of obesity, of particular with the population who consume relatively small amount of dairy products. Second, our data are from a representative nationwide sample that was carefully selected using a systematic sampling method to minimize selection bias and maximize representativeness for the Korean population. Third, there may have been less measurement error for assessment of weight and height because anthropometric measurements were conducted by well-trained health care professionals using standardized equipments. Fourth, we examined dairy product intake using two different dietary assessment method which can assess either long-term (FFQ) or short-term (24-hour recall) intake. Inverse association was found using data from both assessment methods, strengthening our findings.

One of the limitations of our study was that data were obtained from a cross-sectional survey and hence a casual-relationship cannot be proven. In order to minimize the limitation, we excluded data from people who intended to lose weight by changing their diet. We used the representative data but our final analysis population may not be representative of Korean adults because we excluded large number of participants in the analysis.

In conclusion, the results from our study suggest that high consumption of dairy products is related to a lower prevalence of obesity in Korean population. In addition to the findings that Calcium as a component of dairy products may contribute to the inverse association. Prospective studies are warranted to confirm these associations in Asian countries with relatively low intake of dairy products.
